# Managing nitrogen through cover crop species selection in the U.S. mid-Atlantic

**DOI:** 10.1371/journal.pone.0215448

**Published:** 2019-04-12

**Authors:** Jason Kaye, Denise Finney, Charles White, Brosi Bradley, Meagan Schipanski, Maria Alonso-Ayuso, Mitch Hunter, Mac Burgess, Catalina Mejia

**Affiliations:** 1 Department of Ecosystem Science and Management, The Pennsylvania State University, University Park, PA, United States of America; 2 Department of Plant Science, The Pennsylvania State University, University Park, PA, United States of America; 3 Department of Agricultural Production, CEIGRAM, Universidad Politécnica de Madrid, Madrid, Spain; Instituto Agricultura Sostenible, SPAIN

## Abstract

Cover crops have the potential to be agricultural nitrogen (N) regulators that reduce leaching through soils and then deliver N to subsequent cash crops. Yet, regulating N in this way has proven difficult because the few cover crop species that are well-studied excel at either reducing N leaching or increasing N supply to cash crops, but they fail to excel at both simultaneously. We hypothesized that mixed species cover crop stands might balance the N fixing and N scavenging capabilities of individual species. We tested six cover crop monocultures and four mixtures for their effects on N cycling in an organically managed maize-soybean-wheat feed grain rotation in Pennsylvania, USA. For three years, we used a suite of integrated approaches to quantify N dynamics, including extractable soil inorganic N, buried anion exchange resins, bucket lysimeters, and plant N uptake. All cover crop species, including legume monocultures, reduced N leaching compared to fallow plots. Cereal rye monocultures reduced N leaching to buried resins by 90% relative to fallow; notably, mixtures with just a low seeding rate of rye did almost as well. Austrian winter pea monocultures increased N uptake in maize silage by 40 kg N ha^-1^ relative to fallow, and conversely rye monocultures decreased N uptake into maize silage by 40 kg N ha^-1^ relative to fallow. Importantly, cover crop mixtures had larger impacts on leaching reduction than on maize N uptake, when compared to fallow plots. For example, a three-species mixture of pea, red clover, and rye had similar maize N uptake to fallow plots, but leaching rates were 80% lower in this mixture than fallow plots. Our results show clearly that cover crop species selection and mixture design can substantially mitigate tradeoffs between N retention and N supply to cash crops, providing a powerful tool for managing N in temperate cropping systems.

## Introduction

One of the grand challenges for agriculture is to minimize nitrogen (N) losses to the environment while maintaining adequate N supply for high cash crop yields [1]. Cover crops (CCs) are a key strategy for meeting this challenge. Cover crops are typically planted outside of the cash crop growing season to scavenge N from soil and prevent erosion and leaching [2]. Then, when CCs are killed, the N in their tissues can be microbially mineralized to supply inorganic N to subsequent cash crops [3, 4]. While this sounds like an ideal N regulator for agricultural systems, the N dynamics are quite challenging to manage. Cover crops that are good at scavenging N from soil (e.g. grasses) often have high C:N ratios when they are killed. During microbial decomposition of such CC residues, N is immobilized [5, 6], reducing availability to cash crops to an extent that can limit yields in some cases [7–9]. Conversely, legume CC tissues have low C:N ratios and thus, microbes decomposing their tissues mineralize N and increase N availability to cash crops. Unfortunately, these legume CCs can be poor scavengers for soil N and N leaching can be high under them [10–12]. Thus, a major research gap is discovering strategies that enable CCs to prevent N leaching while increasing N supply to and yields of subsequent cash crops. In this paper, we explore CC species selection and CC mixture design as two strategies to improve the balance between N supply and N retention from CCs.

Cover crop adoption is increasing substantially throughout the US [13, 14], and especially in the Mid-Atlantic states [15], including Maryland, Pennsylvania, and Virginia. These US states also have a high concentration of animal agriculture, and it may be possible to couple excess manure from animal production with CC selection to improve N management. Manure has a narrow N:P availability ratio relative to non-leguminous cash crop demand and when manure is applied to meet the N demand of cash crops, excess P can accumulate in soil [16, 17]. Eventually, soil P concentrations become high enough that both erosion and leaching losses ensue, contributing to eutrophication [18]. Thus, it is recommended that farmers add manure to meet the P demand of the crop and then add supplementary N to meet crop demand for N [16]. One way to get this supplementary N is from CCs. This coupling of manure and N from CCs is important in any agroecosystem, but it may be especially valuable in organic grain rotations that rely exclusively on legume and manure N inputs for soil N fertility [19].

To date, most research on balancing N scavenging and supply with CCs has focused on very few species (mainly barley, rye and vetch) and their bicultures [11, 20–23]. These efforts have reported some success in designing bicultures that both limit N leaching and improve N supply to cash crops by increasing the quantity of N supplied and improving the synchrony of N mineralization with crop uptake [24]. However, critical research gaps remain. Studies measuring leaching under legume CCs are rare [10, 11, 25, 26] and many of the CCs that farmers are using have not been studied with respect to the N leaching-N supply tradeoff. The array of CC species that farmers use contrast sharply in mycorrhizal associations (e.g., brassicas do not form symbioses) and winter hardiness, both of which may impact N scavenging from soil and N supply to cash crops [4, 19]. Cover crop selection often varies across rotations, but few experiments have tested the functionality of different CC species in different rotation windows. In addition, studies that simultaneously measure N leaching and N supply in mixtures with more than two species are exceedingly rare [7, 19], even though farmers often use these high-diversity mixtures [27]. Thus, we lack a systematic evaluation of the impacts of CC species selection and high-diversity mixture design on the tradeoff between N retention and N supply in different crop rotation windows.

The goal of this paper is to examine an array of CC species that vary in winter hardiness in our region, mycorrhizal associations, and N fixation to assess how CC selection affects the N cycle and to examine whether mixtures of these CC species help balance tradeoffs between N losses and N supply. For three years we studied six CC monocultures and four mixtures (Table 1) in a maize-soybean-wheat rotation that was managed according to United States Department of Agriculture (USDA) organic standards. The CC treatments were planted in the window between wheat and maize (August planting, long CC window) and in the window between maize and soybean (October planting, short CC window). In previous publications we documented the growth of these CC stands [28] and delivery of a suite of ecosystem services relative to fallow plots [9]. In this paper, we present for the first time the N fluxes in the system, using multiple methods to ask the following questions: 1) How does CC species selection affect N leaching?, 2) How does CC species selection affect N supply to the subsequent maize cash crop?, and 3) Are mixtures superior to monocultures in their potential to balance tradeoffs between N retention and supply to maize? To our knowledge, this work represents the most comprehensive simultaneous assessment of N leaching and N supply for contrasting CCs and especially for high diversity mixtures.

**Table 1:** Cover crop monoculture and mixture seeding rates.

			Number of Species	Clover	Pea	Rye	Oat	Canola	Radish
Treatment	Abbreviation	Next Crop		———————live seeds m^-2^———————					
Fallow control	Fallow		0						
Medium red clover	Clover		1	600					
Austrian winter pea	Pea		1		60				
Canola	Canola		1					400	
Forage radish	Radish		1						60
Oat	Oat		1				300		
Cereal rye	Rye		1			500			
3 species weed	3SppW		3	300		250	150		
3 species nitrogen	3SppN	Maize	3	300	30	100			
3 species nitrogen	3SppN	Soybean	3			250	150		50
4 species	4Spp	Maize	4	300	30	100		200	
4 species	4Spp	Soybean	4		30	100		200	50
6 species	6Spp		6	150	15	100	75	100	20

Republished from [28] under a CC BY license, with permission from American Society of Agronomy Inc, original copyright 2017.

## Materials and methods

### Research site

This study was conducted from 2012–2015 at the Pennsylvania State University Russell E. Larson Agricultural Research Center, Rock Springs, PA (40^o^43’N, 77^o^56’W). Murrill channery silt loam soil (fine-loamy, mixed, semiactive, mesic Typic Hapludult) underlies approximately 80% of the study site, with the remainder consisting of Hagerstown silt loam (fine, mixed, semiactive, mesic Typic Hapludalf) and Buchanan channery loam (fine-loamy, mixed, semiactive, mesic Aquic Fragiudult). Slope is 0–3% on approximately 80% of the site and 3–8% on the remaining area [29]. Surface soil texture (0–20 cm depth) is predominantly clay loam with variability in sand (21.4–27.0%), silt (39.9–48.1%), and clay (29.6–34.3%). Average annual precipitation at the site is 1,020 mm and mean monthly temperatures range from -3°C (January) to 22°C (July) for 1980–2016 [30].

Treatment plots were established in a randomized, full-entry complete block design with four replications. Cash crops were planted in a three-year maize silage-soybean-winter wheat rotation, which is common for Northeast U.S. organic commodity grain farms. Organic management was instituted in July, 2012 and the site received organic certification in 2016. All cash crop seeds were conventional, untreated varieties developed without genetic modification; comparable organic varieties were not available.

Cash crops were planted using commercial-scale equipment in strips 24 m wide by 348 m long (S1 Table has planting and harvesting dates). Dairy bed pack manure from the Pennsylvania State University dairy herd was applied prior to maize and wheat planting at a rate designed to meet the phosphorus (P) requirements of the rotation. Total P demand across all three years of the rotation was projected to be 225 kg P_2_O_5_ ha^-1^ and, due to variability in manure composition, 184 kg P_2_O_5_ ha^-1^ was ultimately applied (S1 Table). Manure was applied on a wet-weight basis at a target rate of 45 Mg ha^-1^ before maize and 34 Mg ha^-1^ before wheat; application rates were not adjusted for moisture- or N-content of the manure. As a result, across the three years, N applied in manure varied from 213 to 395 kg N ha^-1^ prior to maize and from 124 to 164 kg N ha^-1^ prior to wheat. Based on the assumption that 40% of dairy bed pack manure N is available to plants in the year of application [31], plant-available N from the manure addition ranged from 85 to 158 kg N ha^-1^ during the maize growing.

Prior to planting maize, manure was broadcast and within one day was incorporated along with CC residues with a moldboard plow and disc. Prior to planting soybean, CC residues were incorporated with a moldboard plow and disc. To facilitate effective incorporation of CC residues into the soil, CCs were flail mowed prior to plowing. Soybean seeds were treated with *Bradyrhizobium japonicum* inoculum to promote nodulation (N-Dure, Verdesian Life Sciences, Cary, NC). Prior to planting wheat, manure was broadcast and within one day was incorporated along with soybean residue with a chisel plow and disc. Following these primary tillage events, a seedbed was prepared for all crops with an s-tine field cultivator followed by a cultimulcher. Weeds were controlled in the maize and soybean with repeated passes of a tine-weeder, rotary hoe, and inter-row cultivator, as needed. Maize and soybean seeding rates were 82,000 plants ha^-1^ and 444,600 plants ha^-1^ respectively.

Cover crop treatments were planted in 24 m x 29 m split plots within the cash crop strips. Between wheat harvest and maize planting, CCs were established in August (S1 Table contains planting, sampling and termination dates). Cover crops planted between maize harvest and soybean planting were established in late September or early October (S1 Table). Legume seed was inoculated with N-Dure dry inoculant containing the appropriate *Rhizobia* species prior to seeding. The preceding wheat or maize stubble was chisel plowed, disked, S-tined and cultimulched before CC planting. Cover crops were planted with an Almaco (Nevada, IA) Cone Plot Planter mounted on a double disc seed drill (Great Plains Manufacturing, Salina, KS) with 19 cm row spacing [28]. Fallow plots were surface tilled as needed to eliminate weeds in fall and spring, at most once per season.

Cover crop treatments were designed to include both functional and species diversity while meeting specific management objectives. Six monocultures were selected with contrasting functional traits: two legumes (*Fabaceae*), medium red clover (*Trifolium pratense* L.) and Austrian winter pea (*Pisum sativum* L.); two brassicas (*Brassicaceae*), canola (*Brassica napus* L. cv. Wichita) and forage radish (*Raphanus sativus* L. cv. Tillage Radish); and two grasses (*Poaceae*), cereal rye (*Secale cereale* L. cv. Aroostook) and spring oat (*Avena sativa* L. cv. Jerry). One species from each family is known to be winter-hardy in central Pennsylvania (clover, canola, and rye), while the other is known to be susceptible to winter kill (pea, radish, and oat). Each of these species was grown in monoculture at recommended seeding rates (Table 1).

These component species were combined into functional mixtures of increasing species diversity (Table 1). A three-species mixture designed to help manage weeds (3SppW) contained cereal rye, oat, and red clover. A three-species mixture designed to optimize N management (3SppN) differed between planting windows. Prior to maize, the 3SppN mix was clover and pea combined with a low rate of rye to increase N supply to the maize while minimizing N leaching. Prior to soybean, the 3SppN treatment included three N scavengers—rye, oat, and radish—to avoid losses of residual N. A 4Spp mix with greater potential to provision beneficial insects was created by modifying the 3SppN mixes by adding canola in both CC windows, and additionally substituting pea for oat in the window between maize and soybeans. Finally, a 6Spp mix combined each component monoculture into an “insurance mix” with greater functional response diversity and functional effect redundancy. Cereal rye was included in all mixtures due to its reliability, but the rye seeding rate was reduced at higher mix diversity levels. Further details of CC establishment, seeding rates, and mixture design are available in Murrell et al. [28].

Cover crops were terminated by flail mowing within a day of the spring biomass sampling (exact dates in supplemental). All treatments were terminated on the same day. In 2013, CCs were allowed to grow until roughly two weeks before the beginning of the cash crop planting window, by which time cereal rye inflorescences had emerged (Feekes 10.5). This maximized growth of the slower-growing legumes and allowed canola to bloom, providing pollinator resources, but also resulted in very high cereal rye biomass with a high C:N ratio, and subsequent nutrient immobilization (see Results). As a result, in 2014 and 2015 the CCs were terminated 8–9 days earlier, when the rye was in the early or late boot stage (Feekes 10.0).

### Cover crop measurements

Cover crop biomass was sampled as reported in Murrell et al. [28]. Briefly, aboveground biomass taller than ~2 cm was sampled in the fall and spring in three 0.25 m^2^ subplots per plot (S1 Table has sampling dates). Radish roots often protruded more than 2 cm above the soil surface, so radishes were cut at the root-shoot interface to avoid sampling root biomass in only one species. Biomass was sorted to species, dried, weighed, and analyzed for C and N concentrations by the combustion method as described in Finney et al. [7]. A mean value for weed C:N ratio was applied to all weed biomass and included in the calculation of overall C:N for each CC treatment. Due to mechanical cultivation, fallow plots were relatively weed-free and biomass was assumed to be zero, except in experimental weedy subplots as reported by Baraibar et al. [32]. In all other plots, weed biomass was analyzed along with CC biomass since it also affects soil N dynamics.

### Indicators of N leaching potential

Surface soil inorganic N (SIN) concentrations [the sum of ammonium NH_4_^+^ and NO_3_^-^ expressed in mg N kg soil^-1^] were measured beneath CCs to assess the timing and magnitude of treatment effects on surface soil N that could potentially leach. For CCs between wheat and maize, soils were sampled monthly in September, October and November and fortnightly from April until CC termination in mid-May. For CCs between maize and soybeans, sampling was once per month in October, April, and May. At each sampling event, six cores (0.20 m depth by 0.02 m i.d.) were collected in each plot and homogenized. Extractable inorganic N was quantified on a 20 g (fresh weight) subsample extracted with 100 mL 2M KCl and 1 hr of shaking followed by filtration through Whatman 1 filter paper. Extracts were frozen until analysis. Following filtration, remaining soil was sieved to 2 mm to determine the rock fraction of the extracted subsample. A separate 10 g subsample was dried at 105°C for at least 24 hr, weighed, and sieved to 2 mm to determine gravimetric water content of the fresh soil. Extracts were analyzed for NH_4_-N using a microplate colorimetric technique based on the Berthelot reaction [33] and for NO_3_-N using a microplate colorimetric technique based on the Greiss reaction [34].

To quantify the cumulative effects of CCs on the depth distribution of SIN, we collected soil cores to a depth of 80 cm in spring within one week of CC termination and prior to any tillage events. Two cores were collected from each plot, divided into 20 cm increments in the field, and SIN extraction and analysis were conducted as described above for surface soils. Between wheat and maize, samples were collected from all treatments in 2014, and from a subset of treatments in other years. Between maize and soybeans, samples were collected from all treatments except red clover in 2014 and from a subset of treatments in other years. We present data from the year when the most treatments were collected simultaneously, but use all data for regression analyses (see statistics).

Buried anion exchange resin bags were used as an index of cumulative NO_3_-N movement vertically into the subsoil during the CC season [7, 19]. Resin bags were constructed by enclosing 100 mL of moist anion resin beads (Purolite A-400-OH, Res-Kem General Water, Media, PA) in an organza fabric bag and sewing the bag closed to a final dimension of 0.13 m x 0.13 m. The anion exchange capacity of the resin beads was 1.3 eq L^-1^, approximately equivalent to 1.82 g NO_3_-N per bag. Considering the aerial projection of the resin bag, it could theoretically adsorb a loading of 1077 kg NO_3_-N ha^-1^ in percolating soil water. Three bags were buried to a depth of 0.25 m in each plot at the time of CC planting and retrieved immediately prior to CC termination. Upon collection, bags were extracted with 500mL 3M KCl. Extracts were filtered through Whatman 42 filter paper and frozen until analysis. A colorimetric microplate technique based on the Greiss reaction was used to determine the concentration of NO_3_-N in all extracts [34]. The index of cumulative NO_3_-N moving vertically into the subsoil, called “Resin N” in this paper (kg N ha^-1^) was calculated as Resin N = (M_NO3-N_/A_RB_)*10, where M_NO3-N_ is the mass of NO_3_-N accumulated on the membrane in g, A_RB_ is the area of the resin bag (0.0161 m^2^), and 10 converts g N m^-1^ to kg N ha^-1^.

Lysimeters were constructed from 3-gallon plastic buckets, 30.8 cm in diameter, with holes drilled into the bucket lids and covered by 0.75 mm nylon mesh screens. Two buckets were buried in each treatment plot within one entry point of the experiment. The tops of the bucket lids were 35 cm below the soil surface. Two tubes routed through PVC conduit extended from each bucket to the side of the plots. One tube was secured to the bottom of the bucket to suck water out while the second tube returned air to the upper side of the bucket, so no vacuum was created when samples were pumped out of the lysimeter. Lysimeter samples were collected weekly after a significant rainfall event had occurred. A peristaltic pump was connected to the tubing secured to the bottom of the buckets to collect water samples. All water was collected to record volume, and a 20 mL subsample was collected and frozen until analysis for NH_4_-N and NO_3_-N using the same microplate colorimetric technique described above for SIN. We could calculate the flux of N to the lysimeter buckets using the volume and concentration data, but here we present concentration data only because high heterogeneity in water collected in the buckets meant that for a particular rain event some buckets overflowed (precluding a total volume estimate) while others yielded no water. Because it took a full day to sample all lysimeters, some rain events were missed. In winter, snow and freezing temperatures often prevented us from purging the lysimeter buckets, so cumulative losses could not be calculated as they were for resins.

### Indicators of N supply to the maize crop

Cover crops had no impact on soybean or wheat yields [35], so here we focus on CC impacts on N supply to maize. Maize was harvested for silage at 60–70% moisture from two subsamples of crop row at least 5.3 m in length. Samples were taken with at least a 3m buffer from all sides of the plot. Total wet weight was recorded in the field, and silage was chopped in the field and mixed before a 2 L silage subsample was collected, weighed and dried at 60°C to determine moisture content. Subsamples were averaged to represent the entire plot. The oven-dried silage sample was then finely ground and analyzed for N concentration on an elemental analyzer as described for CC biomass [7]. Total N uptake by the maize crop (in kg N ha^-1^) was calculated as the product of the dry silage weight and the N concentration in the silage. Manure was excluded (not spread) from a strip 8 maize-rows-wide (6.1 m) in every plot and we also measured maize silage yield and N concentrations in these manure exclusion plots.

Surface SIN measurements (methods described above) were conducted fortnightly during the maize growing season in both the main manured plot area (6 subsamples per plot) and manure exclusion subplots (4 subsamples per subplot). To represent CC effects on SIN during maize growth, the area under the curve of SIN (SIN_auc_) for the readings between early June and late July was calculated using the auc function in the MESS package in R [36].

In the maize growing season of 2014, we sampled changes in maize height and foliar N concentration to examine synchrony between surface soil SIN (described above) and maize N and growth. Maize height was measured four times between June 18 and August 4 and here we present data from the fallow, pea, rye, and 4Spp treatments. Height was measured on 6 plants per plot. On July 1 and July 18 we used a hole punch to sample tissue in the V5 leaves on 6 maize plants per plot. On August 4, some V5 leaves had senesced so we sampled V7 leaves on all plants. In 2014 and 2015 maize ear leaf samples were also collected when majority of plants were tasseling (VT stage). Tissue samples were ground and analyzed as described above for maize silage.

### Statistical analysis

We used a mixed model with treatment as a fixed effect and block and year as random effects [37]. We initially screened data for potentially important year x treatment interactions, but those were rare and when they occurred they resulted from changes in the magnitude of treatment effects across years, rather than a change in a rank of treatments across years. Thus, our final statistical model did not include year x treatment interactions. Data were transformed as necessary to meet normality and homoscedasticity assumptions. In figures we present the back-transformed estimated marginal means for each treatment. Error for each treatment was calculated by adding and subtracting the standard error of the model (same for all treatments) from each estimated marginal treatment mean (distinct for each treatment) to generate error bounds for transformed data for each treatment. We then back transformed these error bounds and plotted them as upper and lower standard errors in our figures. Because many of our data required log transformations the upper error bar is larger than the lower error bar. We used Fishers LSD for posthoc tests, because in the majority of cases, our interest was in specific treatment contrasts (e.g. fallow vs rye; pea vs the best mixture), rather than examining all possible contrasts. In a few cases (lysimeter N, deep soil SIN), only one year of data was available and we used a mixed model with treatment (fixed) and block (random) effects. For the deep SIN data we analyzed each depth separately and for lysimeter N we analyzed each date separately and then only used dates when all treatments had sufficient (n = 2) replication. To examine relationships among our indicators of N leaching, we used simple linear regression.

## Results

### Nitrogen in CCs between wheat and maize

**Fig 1:**
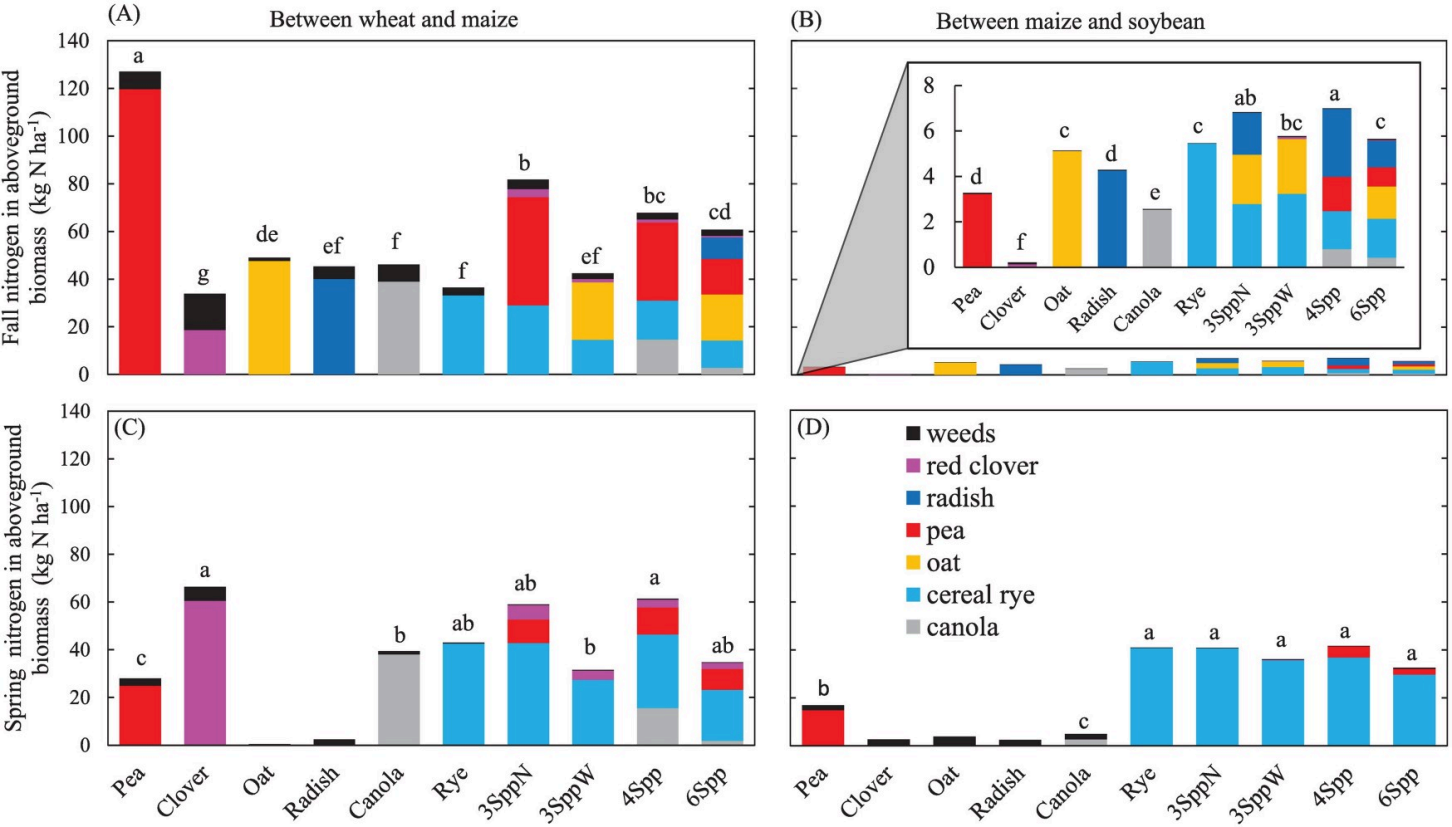
Nitrogen in aboveground cover crop and weed tissues. Values are means (n = 12 replicates) across all three sampling years. Top panels (A and B) are fall biomass and bottom panels (C and D) are spring biomass. Left panels (A and C) are for the cover crop window between wheat and maize and right panels (B and D) are for the window between maize and soybeans. Panel B has an inset on a different scale because of the very low values in this sampling period. Treatments with different lowercase letters had statistically different total cover crop biomass N (Fisher’s LSD, α = 0.05). For spring data (C and D), some species did not over winter, and thus there is no cover crop biomass and no statistical significance reported, though we still plot weed biomass for reference.

Between wheat and maize, non-legume CC monocultures accumulated 30–46 kg N ha^-1^ in fall (Fig 1A), while legume monocultures were either significantly lower (red clover = 17 kg N ha^-1^) or significantly higher (pea = 118 kg N ha^-1^). Mixtures were within the range for monocultures with fall N accumulation roughly proportional to their pea seeding rates; the 3SppW mixture (no pea) accumulated 38 kg N ha^-1^, the 6Spp mixture (15 pea seeds m^-2^) accumulated 57 kg N ha^-1^, and the 3SppN and 4Spp mixtures (both with 30 pea seeds m^-2^) accumulated 64–77 kg N ha^-1^, significantly more than any non-legume monoculture.

In spring (Fig 1C), cover crop biomass N reflected the propensity of species to winter kill. Oat and radish planted between wheat and maize consistently winter killed and did not contain any live biomass N in the spring. Spring N in pea monocultures was highly variable (74, 0, and 0 kg N ha^-1^ in 2013, 2014, and 2015, respectively) because overwintering was inconsistent from year to year. Canola spring N also varied substantially among years (58, 14, and 41 kg N ha^-1^ in 2013, 2014, and 2015, respectively) for the same reason. Across years, clover (55 kg N ha^-1^) and rye (39 kg N ha^-1^) were more consistent in their overwintering, but were not statistically distinct from each other. As with the fall, N in CC biomass of mixtures in spring was proportional to the pea seeding rates, ranging from 29 kg N ha^-1^ (3SppW = no pea), to 32 kg N ha^-1^ (6Spp = 15 seeds m^-2^), to ~54 kg N ha^-1^ (3SppN and 4Spp = 30 seeds m^-2^). It is notable that these mixtures had more spring pea biomass N than pea monocultures, in part because pea overwintered better in mixtures than in monoculture [28].

Between wheat and maize, fall oat stands had the highest C:N ratio (33) and legume monocultures had the lowest (<11) (Table 2). The C:N of rye stands increased between fall sampling (19) and spring sampling (33), while the 3SppW mix had high C:N in both seasons because it was dominated by oat in the fall and rye in the spring. In contrast, canola stands had C:N of ~20 in both fall and spring. Legume stands had C:N of <10 in spring, while all mixtures that contained legumes had C:N of ~22–24 in spring.

**Table 2:** The carbon to nitrogen ratio (C:N) in fall and spring in all aboveground cover crop tissues.

Treatment	Cover crop biomass C:N			
	Between wheat and maize*		Between maize and soybeans**	
	Fall	Spring	Fall	Spring
Pea	11^f^	8^f^	11	10
Clover	11^f^	10^e^	11	11
Oat	33^a^	NA	14	NA
Radish	18^c^	NA	9	NA
Canola	21^b^	19^d^	10	12
Rye	19^bc^	33^a^	13	35
3SppN	13^e^	23^c^	13	32
3SppW	30^a^	29^b^	13	35
4Spp	15^d^	22^c^	11	28
6Spp	21^b^	24^c^	12	31
Error	1	2	NA	NA

*Between wheat and maize, values are estimated marginal means and standard error from the statistical analysis across all three years of study. Within a season, treatments with different lowercase letters had statistically different total cover crop biomass N (Fisher’s LSD, α = 0.05).

**Data not statistically analyzed because in many cases biomass was below our analysis threshold and results are based on too few analyses. These values do not include weed biomass.

NA—Oat and radish winter kill so there are no spring values for these species.

None of the above values include N in weeds. Weeds contained < 5 kg N ha^-1^ in fall except in clover plots, which contained substantially more (11 kg N ha^-1^) (Fig 1A). In spring, weeds contained <2 kg N ha^-1^ in all plots except clover, which was substantially greater (6 kg N ha^-1^). Weeds widened the overall C:N ratio of vegetation in clover plots as detailed in Hunter [38].

### Nitrogen in CCs between maize and soybeans

Between maize and soybeans, all of the CC stands accumulated < 7 kg N ha^-1^ in the fall stands (Fig 1B) and C:N ratios were all relatively low, ranging from 9 to 14 (Table 2). In spring, rye monocultures contained 30–40 kg N ha^-1^, which was significantly higher than any of the other monocultures. The N content of all mixtures was in a similar range, since they were almost entirely composed of rye due to the poor winter-hardiness of the other species when planted in October. Oat and radish winter-killed, and spring biomass N of clover and canola were < 1 kg N ha^-1^. Nitrogen in pea biomass in spring was highly variable from year to year (20, 0, and 23 kg N ha^-1^ in 2013, 2014, and 2015), because winter survival in the window between maize and soybeans was erratic. Weed N was also low in the window between maize and soybeans, both in the fall (< 1 kg N ha^-1^) and the spring (< 2 kg N ha^-1^) (Fig 1B and 1D).

### Potential N losses between wheat and maize

**Fig 2:**
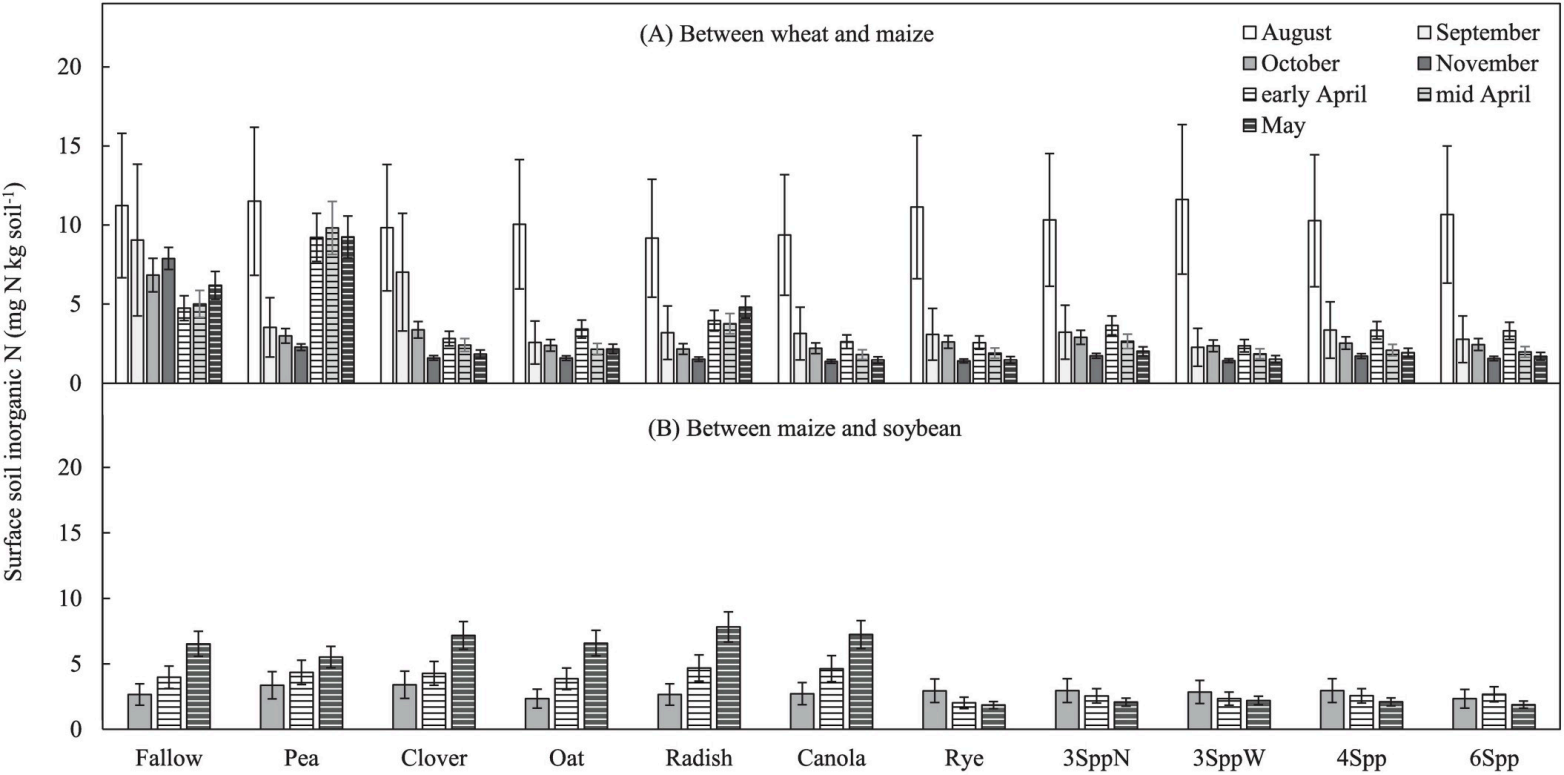
Surface soil inorganic N (SIN) over time. (A) CC window between wheat and maize and (B) between maize and soybeans. Values are back-transformed estimated marginal means and standard error across at least 2 sampling years (4 replicate treatments measured in 2 or 3 years, so n = 8 or 12).

Mean inorganic N concentrations in the surface soils (0–20 cm depth) averaged across all years ranged from 9 to 12 mg N kg soil^-1^ in all treatments immediately after CCs were established in the window between wheat and maize (Fig 2A, S2 Table for statistical details). Surface soils in fallow plots remained in this range until November, while in most CC treatments surface soil inorganic N concentrations declined dramatically between August and September and remained < 5 mg N kg soil^-1^ for the rest of the CC window. Legume monocultures provide two distinct exceptions to this trend as clover plots maintained significantly higher surface soil inorganic N concentrations in September than other CC treatments, and pea monocultures (which winter killed in 2 of 3 years) had significantly higher surface SIN concentrations in April and May than other treatments (Fig 2A, S2 Table). Another exception was radish, which in May had surface SIN (5 ± 1 mg N kg soil^-1^) higher than all treatments other than pea and fallow.

**Fig 3:**
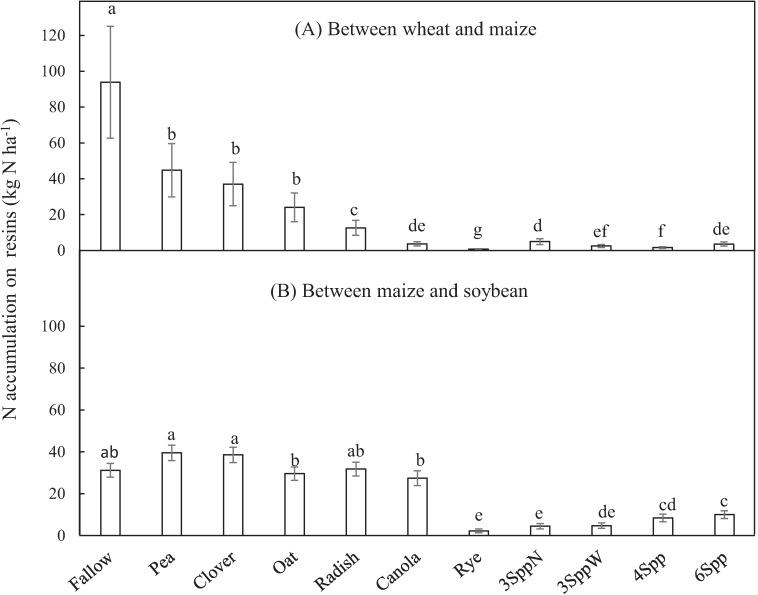
Nitrogen (N) accumulating on resins. Resins buried beneath cover crop treatments planted between (A) wheat and maize or (B) between maize and soybean. Main bars are back-transformed estimated marginal means and error bars are back-transformed model standard error. Three years of data are averaged in (A) (n = 12) and 2 years for (B)(n = 8). Treatments with different lowercase letters had statistically different N accumulation on resins (Fisher’s LSD, α = 0.05).

Mean N accumulating on resins across all years followed the general trend of fallow > legumes > winter-killed non-legume monocultures > mixtures and monocultures containing winter-hardy non-legumes (Fig 3A). Specifically, N accumulating on resins was greatest beneath fallow fields at 94 ± 31 kg N ha^-1^. Mean N on resins under pea and clover legume CCs were 50% less than the fallow, or < 50 kg N ha^-1^. Mean N on resins under oats (24 ± 8 kg N ha^-1^) was not significantly different from the legume monocultures, but radish monocultures were lower (13 ± 4 kg N ha^-1^). Rye had the lowest N accumulation on resins among the treatments (0.7 ± 0.2 kg N ha^-1^), and the other winter-hardy non-legume, canola (4 ± 1 kg N ha^-1^), also had low N accumulation on resins. The mixtures, even those that contained high seeding rates of legumes (e.g. 4Spp and 3SppN), all accumulated between 2 and 5 kg N ha^-1^ on resins.

**Fig 4:**
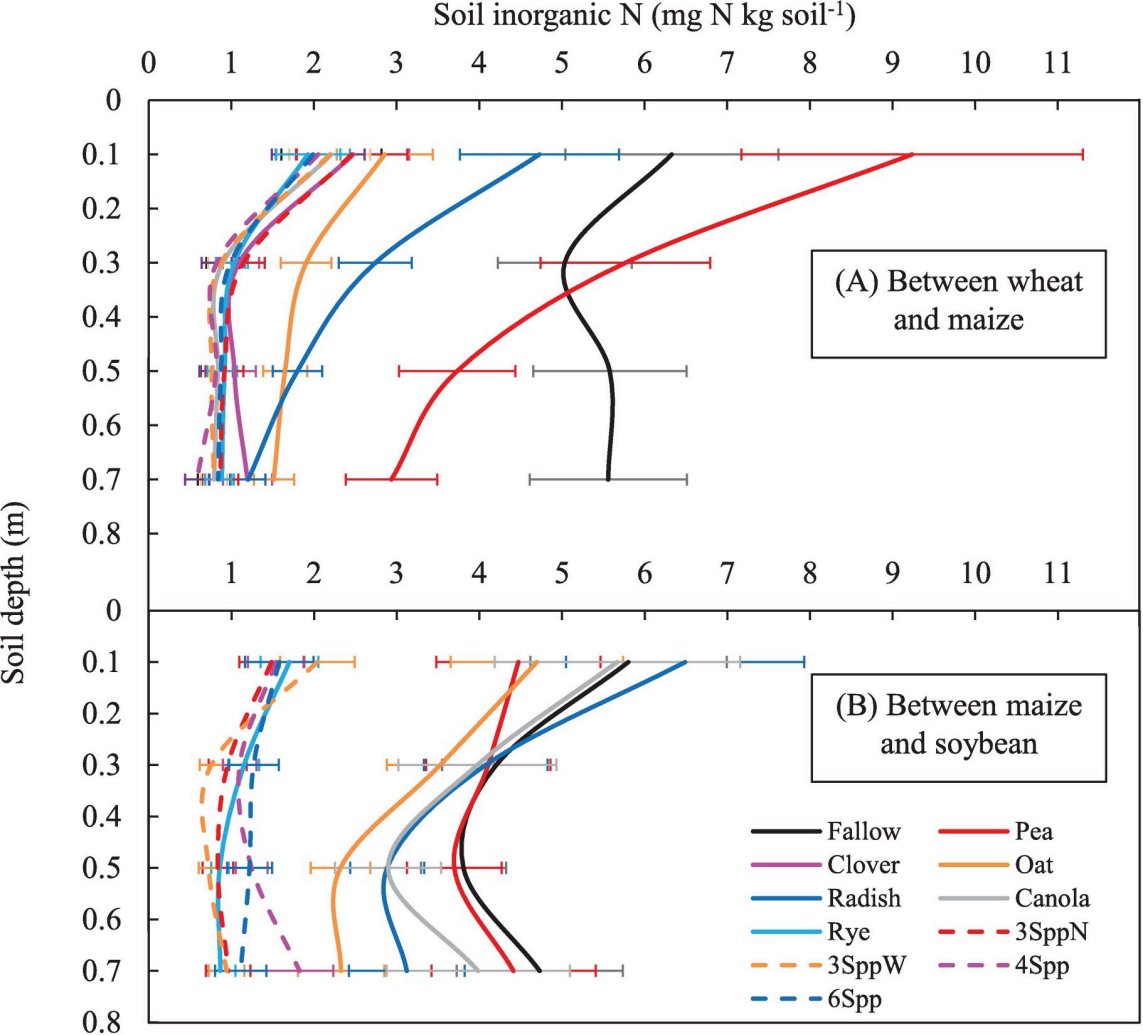
The concentration of extractable soil inorganic N with depth in spring 2014. Soils were sampled at the time of CC termination in the windows between (A) wheat and maize and (B) between maize and soybeans. Values are back-transformed means and standard error of n = 4 replicates.

In this CC window, resin N was significantly correlated with both soil inorganic N concentrations at the 40–80 cm depth in spring (40–80 cm soil N = 0.10*resin N + 1.60; r^2^ = 0.79; p < 0.01; n = 82) and mean lysimeter N concentrations throughout the CC season (average lysimeter N = 0.20*resin N + 2.80; r^2^ = 0.68; p < 0.001; n = 44). Just prior to CC termination in spring, all CCs had significantly less extractable inorganic N than the fallow plots in soils deeper than 40 cm, but pea was not as low as other CC treatments (Fig 4A, S4 Table for statistical details). At the 20 to 40 cm depth, pea and fallow had similar N concentrations in spring, while winter-killed non-legumes (oat and radish) had intermediate values and all other treatments had significantly lower soil N concentrations (Fig 4A, S4 Table).

**Fig 5:**
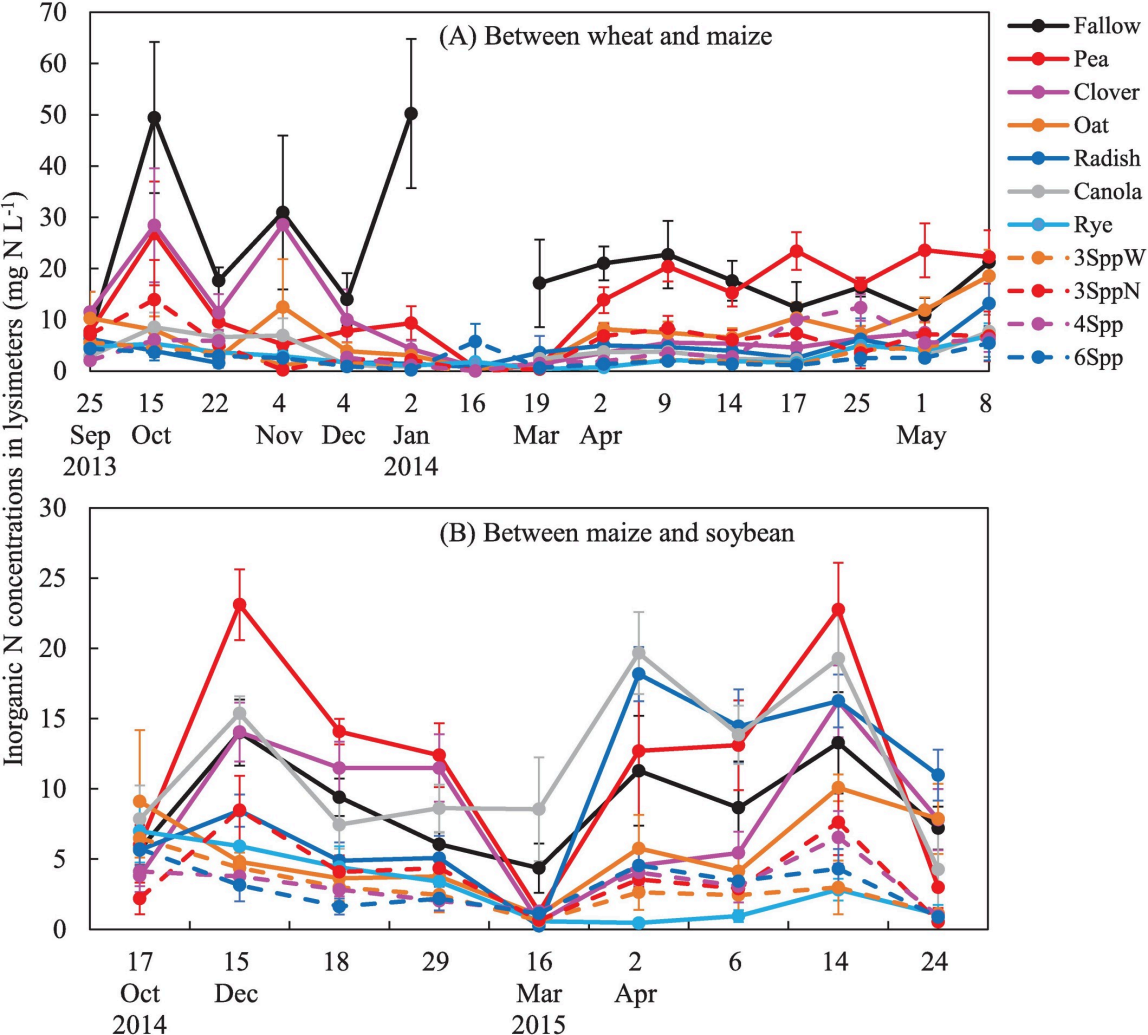
Inorganic N collected in lysimeters beneath all cover crop treatments. Lysimeters were beneath CCs planted between wheat and maize (A) or between maize and soybean (B). Points are means (n = 4) with standard error bars.

Averaged across all sampling dates, inorganic N (mainly nitrate, but presented as nitrate plus ammonium) concentrations in zero-tension lysimeters buried 35 cm below the soil surface were significantly greater in the fallow plots than all other treatments, except pea. However, the key advantage of the lysimeter data (relative to resin data and deep soil coring) is that it provides information about temporal dynamics. Nine of the 15 events that we sampled in lysimeters produced enough water in all treatments to enable statistical analyses of treatment effects on N concentrations (S6 Table). Using these dates, treatment effects can be lumped into four distinct time periods. First, between October and December lysimeter N concentrations in legume monoculture (pea and clover) plots were statistically similar to fallow (and often > 10 mg N L^-1^) while all other treatments were statistically lower than fallow (and < 10 mg N L^-1^) (Fig 5A, S6 Table). Second, all treatments had lower lysimeter N concentrations than fallow on January 2^nd^, and this trend appeared to persist through March, but low replication (due to frozen water in many plots) on other winter sampling dates precluded statistical tests. Third, from April 2^nd^ through April 17^th^ there were several events that produced lysimeter leachate (Fig 5A), and in this spring period, pea monocultures (which winter killed in this sampling year) were statistically similar to fallow (and > 10 mg N L^-1^) while all other treatments (including clover) were statistically lower than fallow (and < 10 mg N L^-1^) (S6 Table). Finally, by May 1^st^, the event closest to the end of the CC season, the lysimeter N concentrations in the fallow plots had declined to levels statistically similar to most other treatments, but pea plots (~20 mg N L^-1^) were statistically higher than all treatments except fallow.

### Potential N losses between maize and soybeans

Mean surface (0 to 20 cm depth) soil inorganic N concentrations were < 5 mg N kg soil^-1^ and statistically similar among all treatments following maize harvest in October and remained low through April (Fig 2B, S3 Table). Between April and May, treatments that did not contain rye increased in soil inorganic N concentrations to 6–8 mg N kg soil^-1^, while all treatments containing rye (rye monocultures and all mixtures) were significantly lower, with surface SIN concentrations < 3 mg N kg soil^-1^.

Mean N accumulating on resins in the window between maize and soybeans was between 25 and 40 kg N ha^-1^ in fallow, clover, pea, oat, radish and canola plots (Fig 3B). In contrast, rye monocultures and all mixtures (because they all contained rye) had significantly lower N accumulation on resins, < 11 kg N ha^-1^. Rye and mixtures that contained rye significantly reduced spring soil inorganic N concentrations relative to fallow at all depths (Fig 4B, S5 Table). Oats and radish also significantly reduced SIN relative to fallow in soils deeper than 40 cm. Inorganic N in the 40–80 cm depth was correlated with resin N (r^2^ = 0.45, p < 0.01) in this CC window, but the 40–60 cm depth had a stronger correlation (40–60 cm soil N = 0.09*resin N + 1.16; r^2^ = 0.67; p < 0.001; n = 76).

Averaging across all events in the window between maize and soybeans, lysimeter N concentrations in rye and all mixtures were statistically lower than fallow, but all other treatments were similar to fallow (Fig 5B, S7 Table). Lysimeter N concentrations were consistently < 10 mg N L^-1^ in rye and mixtures, but > 10 mg N L^-1^ on at least one date in both fall and spring in fallow, pea, clover, radish, and canola treatments (Fig 5B). Temporal dynamics were apparent in oats; they were significantly lower than the treatment with the highest mean concentration (fallow or pea), from December through early April. Then, by April 14^th^, concentrations increased to ~10 mg N L^-1^ in oat plots and they were statistically similar to fallow plots. Mean N concentrations in lysimeters were poorly, but significantly, correlated with resin N accumulation for this CC window (r^2^ = 0.22; p = 0.001).

### Nitrogen supply to maize crops

Cumulative (area under the curve) surface soil inorganic N concentrations during the maize growing season (Table 3) were significantly higher in pea monocultures than most other treatments (the exception is clover) while rye and 3SppW plots had significantly lower SIN than most treatments (oat is an exception). Other mixtures and monocultures were generally in between these endmembers, with few statistical differences among them. Maize ear leaf concentrations ranged from ~2% in rye plots and several mixtures to 3.07% in pea plots (Table 3). Manure exclusion decreased maize ear leaf values by ~ 0.4% but the decrease in ear leaf %N from manure exclusion did not vary among treatments (interaction p = 0.07).

**Table 3:** Indicators of nitrogen (N) availability to the growing maize silage crop across all cover crop treatments.

Cover crop preceding maize	SIN AUC mg N kg soil^-1^	Ear leaf N %	Yield Mg ha^-1^	Silage N %	Silage N kg N ha^-1^
Fallow	593^bc^	2.46^bcd^	35.9^bcd^	0.942^ab^	116.4^cd^
Pea	902^a^	3.07^a^	44.8^a^	0.975^a^	156.3^a^
Clover	796^a^	2.65^bc^	40.1^b^	0.966^a^	137.7^ab^
Oat	499^cd^	2.37^cde^	35.8^cd^	0.908^ab^	110.7^cde^
Radish	652^b^	2.70^b^	39.5^bc^	0.933^ab^	125.8^bc^
Canola	572^bc^	2.49^bcd^	36.0^bcd^	0.816^c^	102.8^de^
Rye	400^e^	2.14^e^	28.6^e^	0.792^d^	80.0^f^
3SppN	590^bc^	2.73^b^	38.7^bc^	0.942^bc^	119.2^bcd^
3SppW	434^de^	2.44^bcd^	32.1^de^	0.825^c^	91.5^ef^
4Spp	631^b^	2.74^b^	37.1^bc^	0.908^ab^	116.5^cd^
6Spp	519^c^	2.34^de^	36.0^bcd^	0.825^c^	101.9^de^
Error	31	0.14	3.9	0.055	9.0

Values are estimated marginal means and standard error from the statistical analysis across all three years of study.

Columns are: the area under the curve (AUC) of repeated measurements of surface (0–20 cm) soil inorganic N (SIN; units mg N kg dry soil^-1^), concentration (%; dry mass basis) of N in maize plant leaves that are adjacent to the ear (i.e. ear leaf), maize silage yield adjusted to 65% moisture, silage N concentration (%; dry mass basis), and N contained in the harvested silage crop.

Within a column (N availability indicator), treatments with different lowercase letters were statistically different (Fisher’s LSD, α = 0.05).

Pea CCs increased subsequent maize silage yield compared to all other treatments (Table 3). In contrast, rye CCs decreased maize yield compared to all other treatments except 3SppW, which contained the highest amount of rye of all the mixture treatments. None of the other mixtures differed from each other or from fallow, oat, canola, radish, or clover monocultures. Manure exclusion consistently reduced yields by 5.9 Mg ha^-1^ but there were no interactions (p = 0.64) between CC treatment and yield impact of excluding manure.

Silage N concentrations following pea and clover were higher than silage N concentrations following the 6Spp, 3SppW, canola, and rye (Table 3). Total N in the maize silage harvest was 156 kg N ha^-1^ following pea CCs, which was significantly higher than all other treatments except clover (Table 3). Maize silage contained 80 kg N ha^-1^ following rye, which was significantly lower than all treatments except 3SppW. Manure exclusion reduced N in maize silage by 26 kg N ha^-1^ and this effect was consistent across CC treatments (interaction p = 0.19).

**Fig 6:**
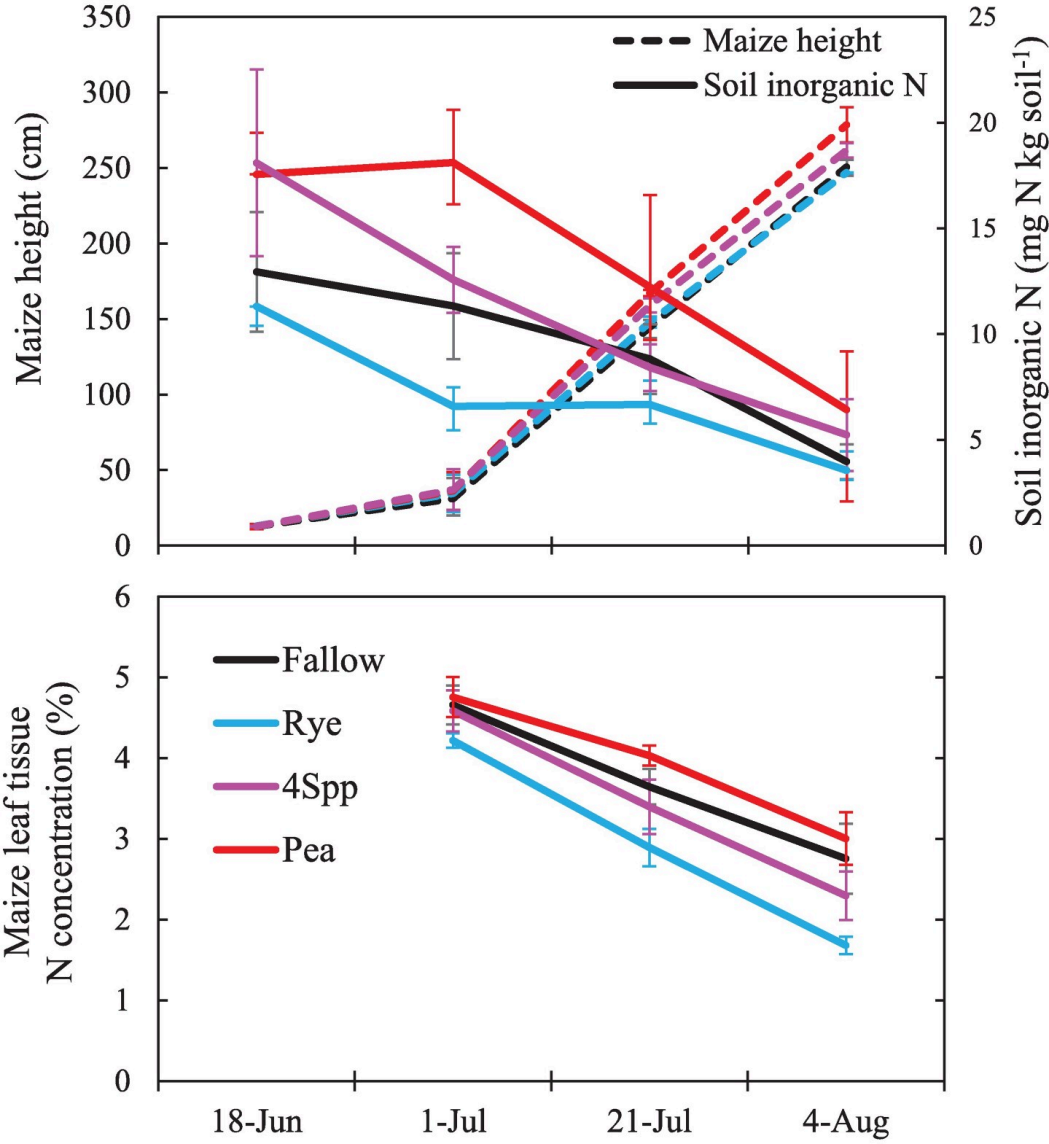
Seasonal progression of maize height (top panel; left axis), surface soil inorganic N concentrations (top panel; right axis), and maize leaf N concentrations (bottom panel) in four cover crop treatments in 2014. Points are means (n = 4) and one standard error.

In 2014, we intensively sampled maize growth dynamics along with soil N availability in a subset of plots (Fig 6). Soil sampled just after maize planting (June 18) had high SIN (18–20 mg N kg soil^-1^) in pea monocultures and 4Spp plots but they were not significantly different from fallow and rye plots (S8 Table). By early July, pea monocultures (which winter killed in this sampling year) had significantly higher SIN than other treatments and the 4Spp treatment had higher SIN than rye. As maize growth accelerated (as indicated by changes in height), SIN declined in all treatments so that by the end of July they were all statistically similar and between 4 and 8 mg N kg soil^-1^ (Fig 6). Treatments had statistically similar maize heights on June 18 (V3 stage), but at the V5 stage (July 1) and V9 stage (July 21) the pea and 4Spp were significantly taller than fallow, and by VT (August 4), pea was significantly taller than rye and fallow. Nitrogen concentrations in maize leaves were higher in pea than in rye on all sampling dates and fallow and 4Spp treatments were intermediate and generally not significantly different from either pea or rye (Fig 6, S8 Table).

## Discussion

### How does CC species selection affect N leaching?

In the CC window between wheat and maize, all CC species reduced N leaching potential relative to the fallow. This assertion is supported by an array of independent measurements across three years showing that all CCs reduced surface soil inorganic N concentrations in the fall and spring; CCs reduced the concentrations of N in lysimeters either in fall, winter, spring or all seasons; all CCs reduced N accumulating on resins over the entire CC growing season; and all CCs reduced concentrations of N in deep soil layers in spring. While it was fully expected that rye CCs would reduce N leaching, most of the other species in our experiment had not been thoroughly tested for their ability to prevent N leaching, and results have been variable in prior legume and mixture CC leaching studies [12]. Thus, our work represents strong evidence that the practice of cover cropping, regardless of species and including legumes, can have positive impacts on water quality.

While all CCs reduced N leaching, the N dynamics under each species are important to consider for CC system design. Legume CCs mitigated N leaching during the CC season, but they had the smallest leaching reduction relative to fallow. Differences in the phenology of the CCs were also evident. Pea grows rapidly in fall and as a result it reduced soil inorganic N in the fall and lysimeter N leaching was generally low in the winter in this treatment. However, pea dies back substantially in winter in our area, and as soils warmed, mineralization of dead pea biomass increased SIN and lysimeter N concentrations in spring. Compared to pea, clover grew more slowly in fall and did not draw down fall SIN as rapidly, but the treatments had statistically similar fall N concentrations in lysimeter water. This is likely because the clover stands were poorly competitive, so weeds proliferated [32] and accumulated substantial N in their biomass. We suspect that high weed biomass was important in allowing the clover plots to have low lysimeter N concentrations in spring. White et al. [19] came to a similar conclusion from a multi-year on-farm research study showing that increasing levels of fall weed biomass reduced N leaching in clover CC stands. Notably, at one farm with high soil organic matter (and associated N mineralization), clover CC stands with low weed biomass had substantial N leaching beneath the CC [19].Thus, we recommend further research on legume-based CCs to determine the conditions under which they reduce N leaching.

Winter-killed non-legumes also showed important seasonal dynamics. Radish and oats grew rapidly and accumulated substantial biomass N in fall, keeping surface SIN levels and lysimeter N concentrations low. But after winter killing, N cycling from the dead biomass was evident in increased surface SIN levels and lysimeter N concentrations in late spring (May). Thus, integrated over the growing season, these CCs had intermediate levels of N accumulating on buried resins and intermediate levels of SIN at the 20–40 cm depth. Interestingly, the aboveground biomass N accumulation in radish and oat monocultures (~50 kg N ha^-1^) was less than the reduction in resin N (65 to 75 kg N ha^-1^) these CCs achieved relative to fallow. This suggests that some of the N retention by these CCs was due to uptake into root biomass or CC-stimulated microbial N immobilization. As expected, the lowest N leaching level (by all measures) occurred in plots containing rye, which accumulated substantial N in fall, then over wintered to accumulate even more N in biomass in spring. The reduction in resin N by rye (~80 kg N ha^-1^ relative to fallow) was comparable to the N mass in fall plus spring aboveground biomass in the rye monocultures.

One of our goals was to determine whether mixtures reduced leaching as well as non-legume monocultures. In the window between wheat and maize the biomass of mixtures in the fall was fairly evenly represented by all the planted species, while in spring all of the mixtures were dominated by rye [28]. By all measures, mixtures performed better than the fallow and legume monoculture plots, so mixing in non-legumes, even at low seeding rates, appears to have a substantial impact on potential N leaching. For example, N accumulating on resins was ~70% lower in mixtures than monocultures other than rye. Yet, temporal dynamics in the mixtures also seem to reflect the influence of the legumes in those mixes. For example, the 4Spp mix periodically had high lysimeter N values in spring (≥ 10 mg N L^-1^ on April 17^th^ and May 1^st^, though not statistically different from other mixtures), which may reflect mineralization of senesced pea or canola residues in those stands. Nevertheless, integrated over the growing season, the mixtures accumulated very little N on resins, and by spring all of the mixtures had low SIN concentrations in the deep soil. Thus, in this CC window there is strong potential for mixtures, even with low seeding rates of non-legumes, to confer large reductions in N leaching relative to fallow plots or legume monocultures.

In the CC window between maize and soybeans, the soils started with quite low SIN, and N accumulation was lower on resins when compared to the window between wheat and maize. This is likely due to limited opportunities for N mineralization and leaching between planting in October and soil freezing. With this late planting date, only rye established well and also over-wintered. Pea overwintered in 2 of 3 years, but always had low biomass. All other CC species had limited fall growth and then died over winter [28]. Thus, rye was the only monoculture that conclusively reduced N leaching relative the fallow plots in this window. The reduction in resin N by rye in this window was ~30 kg N ha^-1^ relative to fallow, while N in fall plus spring aboveground biomass was ~40 kg N ha^-1^.

Still, there is some indication that oats reduced N leaching in the window between maize and soybeans, particularly in the fall. Oat monocultures had fall biomass N and winter lysimeter N concentrations comparable to rye, and at the end of the CC season, SIN in deep soil layers (40–60 cm and 60–80 cm) was lower in oat plots than fallow plots, suggesting less downward transport of N. It seems likely that N accumulating on resins beneath the oat plots resulted mainly from spring transport, when both surface SIN and lysimeter N concentrations increased. The patterns described above for oats also occurred in radish plots, though they were rarely significant.

Results from the window between maize and soybean are interesting because they suggest that even when CCs are planted late in the fall, they still produce measurable changes in some N leaching indicators. This result is consistent with our prior model simulations showing that low-biomass, late-planted cover crops reduce N leaching [2], but it contrasts with conventional wisdom. We expected that CCs other than rye would have limited effects on N leaching when planted after maize silage due to low biomass production potential of most other species with late fall planting dates. While our results indicate that rye is clearly the best monoculture, they also show that other species can reduce N leaching. It is possible that with slightly earlier planting dates (e.g. September) or in locations with slightly warmer fall temperatures (or later frost dates) oat, and perhaps radish, canola, and pea, could be effective at reducing N leaching after maize silage. For these CCs at the edge of their functional range, mixtures may be a good way to ensure CC performance. In the window between maize and soybeans, mixtures substantially outperformed many monocultures because rye (a reliable late-season performer) was a component of all of our mixtures. Seeding even a small rate of rye turned the CC mixture into effective N scavengers. This suggests that with precariously late planting dates or variable winter climate, adding a small seeding rate of a known performer, such as rye, into the CC mix could be a valuable insurance approach for designing mixes that consistently reduce N leaching. Many farmers are also interested in reducing seeding rates of rye monocultures in order to save on seed costs. Our results from the maize-to-soybean CC window support the idea that seeding rates of rye could be reduced without significantly compromising the effectiveness of N scavenging.

### How does CC species selection affect N supply to the subsequent maize crop?

Previous research by our team showed that the biomass N and the C:N ratio of the CC can influence yield of subsequent cash crops [3, 4, 7, 39]. Indeed, some of the data from the current study were included in models developed by White et al. [19] that predicted the maize yield response to N mineralization from cover crop residues. Here we use additional and more frequent measures of N dynamics across a longer duration experiment to increase understanding of the mechanistic link from CCs to yield through changes in soil N and maize tissue %N. Following pea monocultures, soil inorganic N is high near the maize planting date and remained high well into the maize growing season. This led to higher N concentrations in maize leaves following legume CCs, especially during the part of the season when maize accumulated height rapidly. At the end of the growing season, maize in plots with preceding pea monocultures were taller, had higher N concentrations in tissues, and had higher yield than other CC treatments even in years when pea winter killed.

We applied manure at a rate to meet the P demand of the crop. For farmers with high P soils, this would be the maximum amount of manure that could be added to a field while still meeting nutrient management regulations in the mid-Atlantic [40]. However, with dairy manure, applications meeting the P demand of a maize crop may not supply sufficient N to meet the N demand of that crop [16]. We expected that N from legume CCs would supplement manure N to optimize yields. Comparing N uptake in manured and manure exclusion plots across treatments gives us some estimates of the direct impact of manure and legume N sources on maize. The ~300 kg N ha^-1^ of manure we added increased N uptake by an average of 26 kg N ha^-1^ across all CC treatments, while planting pea increased N uptake (relative to fallow) by ~40 kg N ha^-1^. The combination of manure plus a pea monoculture increased N uptake by ~70 kg N ha^-1^ relative to manure-exclusion subplots in the fallow treatment. If farmers used nutrient supplements instead of cover crops to increase N supply, it would cost 60 to 120 US dollars ha^-1^ (20 to 50 USD acre^-1^) of inorganic synthetic N fertilizer or > 600 US dollars ha^-1^ (>240 US dollars ac^-1^) of N-rich organic fertilizer to get the same increase in N uptake that our pea CC generated (assuming a plant fertilizer recovery of 50%, with price ranges coming from variation in fertilizer prices). For comparison, Austrian winter pea seeds currently costs 170 US dollars ha^-1^ (70 US dollars ac^-1^). It is unclear from our data whether additional manure or more productive legume CCs would have increased yields to higher levels than we observed in our pea plots. Ear leaf N concentrations suggest that N availability may have been sufficient in the manured pea plots (> 2.75% [41]), but was below sufficiency levels in all other plots. However, we did not observe an interaction between CC treatment and the manure effect on N uptake, which might have occurred if pea CCs were providing so much N that the effect of manure exclusion was lower in pea plots than the fallow plot (or other CC treatments).

In contrast to legume monocultures, rye monocultures negatively impacted yield of the subsequent maize crop, relative to fallow, through changes in N supply. Rye plots accumulated substantial N, but in wide C:N ratio tissues. After observing this phenomenon in the first year of the study, we killed the CCs earlier [28], which reduced rye biomass and C:N ratio at termination [38]. Even with this tweak, however, maize crops that followed rye monoculture CCs had low SIN at maize planting and throughout the maize growing season, leading to low concentrations of N in maize tissues and low yields. Aboveground N uptake in rye plots was 40 kg N ha^-1^ lower than in fallow plots (comparing manure exclusion plots only), providing a direct estimate of the N immobilization impacts of rye on maize. This result has been reported in prior studies [7] and since rye is widely used, CC outreach should emphasize the yield risks of rye in N-limited sites. Increasing soil organic matter can mitigate the negative impacts of wide C:N CCs on yield [19], as can supplemental fertilizer [42].

All other CC monocultures fell between the two endmembers of pea and rye in their impacts on the N dynamics during the maize growing season and the impacts of those dynamics on yields. However, contrasting temporal dynamics were evident across the monoculture treatments. For example, oats accumulated just as much fall biomass N as rye, and the C:N ratio was quite wide, but because oats winter killed, there was ample time for mineralization [4] before maize was planted, so oats did not negatively impact yields (relative to fallow) in the same way rye did. Mixtures had yields and N uptake comparable to the fallow plots, suggesting that mixing in legumes while reducing rye seeding rates ameliorates the negative impacts of rye on yield.

### Are mixtures superior to monocultures in their potential to balance tradeoffs between N retention and supply?

We previously reported strong tradeoffs among 7 ecosystem services in both monocultures and mixtures in this experiment [9]. That work found that pea monocultures were the best treatment for balancing these tradeoffs, but that compared to the other 5 monocultures, mixtures increased multifunctionality by minimizing ecosystem disservices. In Finney et al. [9], we compared ecosystem service values that were first relativized to fallow plots (CC plot minus fallow plot = ecosystem service) and then divided by their standard deviation to put values on a comparable scale. This approach was essential for comparing 7 diverse ecosystem services in the same analysis, but it limited our ability to evaluate real fluxes of N. In this paper, with our detailed, multi-method, multi-year N analysis of the same system, we find a very skewed impact of mixtures: they impact N leaching much more than N supply. Compared to the best-yielding CC treatment (pea), mixtures were only 15% lower in yield, but compared to the worst-leaching plot (fallow) the mixtures were 90% better. When using a single monoculture CC as the benchmark for tradeoffs, no monoculture did as well as mixtures: pea had better yields, but worse N leaching than mixtures; oat and radish had the same yield as fallow and the mixtures, but had more leaching than the mixtures; rye had lower yields than mixtures, but only slightly less N leaching. If our benchmark for tradeoffs is a fallow plot, then mixtures were clearly effective in balancing tradeoffs–mixtures had the same yield as the fallow, but had much less N leaching.

All of these comparisons, and the skewness of the relationships, point to the fact that “tradeoffs” is a value-laden concept. What yield impact is acceptable to achieve low N leaching? What level of N leaching reduction is considered a success? We had hoped to find win-win situations with no tradeoffs; to discover CC mixtures with yields that were not significantly different from the highest yielding plots (in this case pea), and leaching that is as low as the lowest plots (rye). We did not achieve that goal; however, our measures of N dynamics point to a path forward in new mixture design. Mixtures certainly performed well on the N leaching side; reducing leaching to within a few kg N ha^-1^ of the rye plots. Thus, a key outcome from our work is that a low seeding rate (Table 1) of winter-hardy non-legumes can reduce leaching relative to fallow fields or legume monocultures. Our mixtures did not produce the highest-yielding maize, but we hypothesize that can be achieved through both mixture design and improved soil organic matter dynamics. Building soil organic matter could make yields more resilient to N immobilization during CC decomposition [19]. Moreover, future mixture designs may be able to reduce rye and increase legume biomass to increase N supply from CC mineralization without substantially increasing N leaching. Finally, in assessing tradeoffs, it is important to consider that the small yield reduction in mixtures (relative to fallow) could be reversed with N fertilizer, whereas N leaching cannot be as effectively combatted through any other management strategy or purchased input. It may be better to use CCs to maximize the service that cannot be purchased elsewhere (reductions in N leaching), but to do so in a way that does not cause an undue burden on yields. From this perspective, mixtures provide a powerful tool to optimize cropping systems using integrated nutrient management.

## Conclusions

We observed substantial variation among CC monocultures in N dynamics including biomass N accumulation, N leaching, and N supply to a maize cash crop. Thus, CC selection can be a valuable tool for N management. A key result is that all CCs reduced leaching relative to fallow plots, including legumes and winter-killed non-legumes. We found that pea monocultures increase maize yields in comparison with fallow or non-legume monocultures and that they increase maize N uptake even more than our large manure application. However, non-legume CCs, especially winter-hardy grasses, need to be managed carefully so that large stands of high C:N CCs do not lead to N limitation in subsequent maize crops.

The mixtures we tested had positive N management outcomes in that they substantially reduced leaching losses while maintaining maize yields comparable to fallow plots. Our results revealed a strong skewness of CC mixture impacts on N: mixtures impacted N retention substantially more than N supply to maize crops. This result points to great potential for mixtures as an N management tool, though improvements in mixture design could still be realized. Maize following mixtures yielded lower than maize following pea plots because rye dominated all of our mixtures in spring, and because red clover was a poor competitor in mixtures [28]. We are now testing new mixtures that include triticale and crimson clover as substitutes for rye and red clover, respectively. We are also developing models that allow us to predict how much N mineralization from soil and CC residues will become available to maize crops, which should improve recommendations for supplemental manure additions in organic systems like ours, or synthetic fertilizer N addition recommendations for conventionally managed systems [4, 19]. With continued testing of CC mixtures, and models that link them to soil and cash crop N, mixtures could become an important N regulator, enabling high yields with low supplemental N inputs and low N leaching.

## Supporting information

S1 Table Field management details. Adapted from Hunter 2018 [38].
(DOCX)

S2 Table Statistical results for surface soil inorganic nitrogen (SIN) data for cover crops grown between wheat and maize. Different letters denote statistical differences among cover crop treatments (rows) for a given time period (columns) based on Fishers LSD and α = 0.05. Statistical tests were conducted across all three years of the experiment. See Table 1 for treatment codes.
(DOCX)

S3 Table Statistical results for surface soil inorganic nitrogen (SIN) data for cover crops grown between maize and soybeans. Different letters denote statistical differences among cover crop treatments (rows) for a given time period (columns) based on Fishers LSD and α = 0.05. Statistical tests were conducted across all three years of the experiment. See Table 1 for treatment codes.
(DOCX)

S4 Table Statistical results for soil inorganic nitrogen (SIN) data from cover crops grown between wheat and maize. These deep soil SIN samples were collected on one date in spring 2014 near the date of cover crop termination. Different letters denote statistical differences among cover crop treatments (rows) for a given soil depth (columns) based on Fishers LSD and α = 0.05. See Table 1 for treatment codes.
(DOCX)

S5 Table Statistical results for soil inorganic nitrogen (SIN) data from cover crops grown between maize and soybeans. These deep soil SIN samples were collected on one date in spring 2014 near the date of cover crop termination. Different letters denote statistical differences among cover crop treatments (rows) for a given soil depth (columns) based on Fishers LSD and α = 0.05. See Table 1 for treatment codes.
(DOCX)

S6 Table Statistical results for bucket lysimeter inorganic N data for cover crops grown between wheat and maize. Different letters denote statistical differences among cover crop treatments (rows) for a given time period (columns) in 2013 (October and December) and 2014 (all other dates) based on Fishers LSD and α = 0.05. See Table 1 for treatment codes.
(DOCX)

S7 Table Statistical results for bucket lysimeter inorganic N data for cover crops grown between maize and soybeans. Different letters denote statistical differences among cover crop treatments (rows) for a given time period (columns) in 2014 (October and December) and 2015 (all other dates) based on Fishers LSD and α = 0.05. See Table 1 for treatment codes.
(DOCX)

S8 Table Statistical results for changes in soil inorganic N, maize leaf tissue N concentration, and maize height in 2014. Different letters denote statistical differences among cover crop treatments (rows) for a given sampling date (columns) in 2014 based on Fishers LSD and α = 0.05. See Table 1 for treatment codes.
(DOCX)
