# Supplementary material for: Managing nitrogen through cover crop species selection in the U.S. mid-Atlantic
Source: PLoS One. 2019 Apr 12;14(4):e0215448. doi: 10.1371/journal.pone.0215448 (PMC6461281; doi:10.1371/journal.pone.0215448)
Supplement: S1 Table — Adapted from Hunter 2018 [38]. (DOCX) [file pone.0215448.s001.docx]

**S1 Table. Field management details.** Adapted from Hunter 2018.

|  |  | Timing of Field Operations and Sampling | | | | | |  | |  | |  | |  |
| --- | --- | --- | --- | --- | --- | --- | --- | --- | --- | --- | --- | --- | --- | --- |
| Crop ^a^ | Year | Seed Cover Crop | Cover Crop Sampling Dates | | Apply Manure | Plant | Harvest | Crop Seeding Rate | Dry Manure Rate | | Manure N | | Manure Phosphate | |
|  |  |  | Fall | Spring |  |  |  | seeds ha^-1^ | Mg ha^-1^ | | kg N ha^-1^ | | kg P_2_O_5_ ha^-1^ | |
| Maize | 2012 | 8/25 | 11/9 |  |  |  |  |  |  | |  | |  | |
|  | 2013 | 8/8 | 10/29 | 5/13 | 5/15 | 5/31 | 9/10 | 82,000 | 15.7 | | 213 | | 75.0 | |
|  | 2014 | 8/15 | 11/5 | 5/5 | 5/8 | 6/2 | 9/15-16 | 82,000 | 20.7 | | 353 | | 76.6 | |
|  | 2015 |  |  | 5/4 | 5/11 | 5/28 | 9/14-15 | 82,000 | 17.7 | | 395 | | 131 | |
| Soybean | 2012 | 10/10 | 11/19 |  |  |  |  |  |  | |  | |  | |
|  | 2013 | 9/20 | 11/5 | 5/21 |  | 6/5 | 10/02 | 444,600 |  | |  | |  | |
|  | 2014 | 9/30 | 11/12 | 5/13 |  | 6/3 | 10/14 | 444,600 |  | |  | |  | |
|  | 2015 |  |  | 5/13 |  | 6/10 | 10/14 | 444,600 |  | |  | |  | |
| Wheat | 2012 |  |  |  | 10/23 | 10/25 |  | 4,500,000 | 12.3 | | 164 | | 88.8 | |
|  | 2013 |  |  |  | 10/3 | 10/16 | 7/15-16 | 4,500,000 | 12.1 | | 137 | | 66.0 | |
|  | 2014 |  |  |  | 10/21 | 10/24 | 7/21 | 5,700,000 | 12.0 | | 124 | | 114 | |
|  | 2015 |  |  |  |  |  | 7/22 |  |  | |  | |  | |

^a^ Maize hybrid: Master’s Choice MC4050 maize in all years; soybean variety: Growmark FS HS 22C10 in 2013 and 2014 and HS 21C40 in 2015; wheat variety: cv. Malabar; W.I. Miller and Sons, Farmdale, OH.

Hunter, M. 2018. Sustainable Intensification and Climate Resilience: Cover Crops, Soil Improvement, and Drought. Doctoral Dissertation, Penn State University.
