# Supplementary material for: Managing nitrogen through cover crop species selection in the U.S. mid-Atlantic
Source: PLoS One. 2019 Apr 12;14(4):e0215448. doi: 10.1371/journal.pone.0215448 (PMC6461281; doi:10.1371/journal.pone.0215448)
Supplement: S2 Table — Different letters denote statistical differences among cover crop treatments (rows) for a given time period (columns) based on Fishers LSD and α = 0.05. Statistical tests were conducted across all three years of the experiment. See Table 1 for treatment codes. (DOCX) [file pone.0215448.s002.docx]

**S2 Table. Statistical results for surface soil inorganic nitrogen (SIN) data for cover crops grown between wheat and maize.** Different letters denote statistical differences among cover crop treatments (rows) for a given time period (columns) using Fishers LSD (α = 0.05). Statistical tests were conducted across all three years of the experiment. See Table 1 for treatment codes.

| Treatment | August | September | October | November | Mid March | Early April | Mid April | Early May | Mid May |
| --- | --- | --- | --- | --- | --- | --- | --- | --- | --- |
| Fallow | a | a | a | a | b | b | b | b | c |
| Pea | a | b | bc | b | a | a | a | a | a |
| Clover | ab | a | b | c | c | d | c | de | d |
| Oat | a | bc | c | c | c | cd | e | d | d |
| Radish | b | b | c | c | c | cd | c | c | b |
| Canola | b | b | c | c | c | e | e | e | e |
| Rye | a | b | c | c | c | e | e | e | e |
| 3SppN | ab | b | bc | c | c | d | de | d | d |
| 3SppW | a | c | c | c | c | e | de | e | e |
| 4Spp | ab | b | c | c | c | d | e | d | d |
| 6Spp | ab | bc | c | c | c | cd | e | de | de |
