# Supplementary material for: Managing nitrogen through cover crop species selection in the U.S. mid-Atlantic
Source: PLoS One. 2019 Apr 12;14(4):e0215448. doi: 10.1371/journal.pone.0215448 (PMC6461281; doi:10.1371/journal.pone.0215448)
Supplement: S3 Table — Different letters denote statistical differences among cover crop treatments (rows) for a given time period (columns) based on Fishers LSD and α = 0.05. Statistical tests were conducted across all three years of the experiment. See Table 1 for treatment codes. (DOCX) [file pone.0215448.s003.docx]

**S3 Table. Statistical results for surface soil inorganic nitrogen (SIN) data for cover crops grown between maize and soybeans.** Different letters denote statistical differences among cover crop treatments (rows) for a given time period (columns) using Fishers LSD (α = 0.05). Statistical tests were conducted across all three years of the experiment. See Table 1 for treatment codes.

| Treatment | October | Early April | May |
| --- | --- | --- | --- |
| Fallow | ab | a | a |
| Pea | a | a | a |
| Clover | a | a | a |
| Oat | b | a | a |
| Radish | ab | a | a |
| Canola | ab | a | a |
| Rye | ab | bc | b |
| 3SppN | ab | d | b |
| 3SppW | ab | cd | b |
| 4Spp | ab | d | b |
| 6Spp | b | d | b |
