# Supplementary material for: Managing nitrogen through cover crop species selection in the U.S. mid-Atlantic
Source: PLoS One. 2019 Apr 12;14(4):e0215448. doi: 10.1371/journal.pone.0215448 (PMC6461281; doi:10.1371/journal.pone.0215448)
Supplement: S5 Table — These deep soil SIN samples were collected on one date in spring 2014 near the date of cover crop termination. Different letters denote statistical differences among cover crop treatments (rows) for a given soil depth (columns) based on Fishers LSD and α = 0.05. See Table 1 for treatment codes. (DOCX) [file pone.0215448.s005.docx]

**S5 Table. Statistical results for soil inorganic nitrogen (SIN) data from cover crops grown between maize and soybeans.** These deep soil SIN samples were collected on one date in spring 2014 near the date of cover crop termination. Different letters denote statistical differences among cover crop treatments (rows) for a given soil depth (columns) using Fishers LSD (α = 0.05). See Table 1 for treatment codes.

| Treatment | 20-40 cm | 40-60 cm | 60-80 cm | 40-80 cm | 0-80 cm |
| --- | --- | --- | --- | --- | --- |
| Fallow | ab | a | a | a | a |
| Pea | b | a | ab | ab | ab |
| Clover^#^ | n/a | n/a | n/a | n/a | n/a |
| Oat | ab | b | c | cd | b |
| Radish | a | b | bc | bc | ab |
| Canola | ab | b | ab | abc | ab |
| Rye | c | cd | e | f | d |
| 3SppN | c | cd | e | f | c |
| 3SppW | c | d | e | f | c |
| 4Spp | c | c | d | de | c |
| 6Spp | c | cd | e | ef | c |

^#^No samples were collected from clover plots in this cover crop window.
