# Supplementary material for: Managing nitrogen through cover crop species selection in the U.S. mid-Atlantic
Source: PLoS One. 2019 Apr 12;14(4):e0215448. doi: 10.1371/journal.pone.0215448 (PMC6461281; doi:10.1371/journal.pone.0215448)
Supplement: S6 Table — Different letters denote statistical differences among cover crop treatments (rows) for a given time period (columns) in 2013 (October and December) and 2014 (all other dates) based on Fishers LSD and α = 0.05. See Table 1 for treatment codes. (DOCX) [file pone.0215448.s006.docx]

**S6 Table. Statistical results for bucket lysimeter inorganic N data for cover crops grown between wheat and maize.** Different letters denote statistical differences among cover crop treatments (rows) for a given time period (columns) in 2013 (October and December) and 2014 (all other dates) using Fishers LSD (α = 0.05). See Table 1 for treatment codes.

| Treatment | Season average | Oct 15th | Oct 22^nd^ | Dec 4^th^ | Jan 2^nd^ | April 2^nd^ | April 9^th^ | April 14^th^ | April 17^th^ | May 1st |
| --- | --- | --- | --- | --- | --- | --- | --- | --- | --- | --- |
| Fallow | a | a | a | a | a | a | a | a | ab | abc |
| Pea | ab | abc | ab | ab | b | ab | a | a | a | a |
| Clover | bc | ab | ab | ab | bc | cd | bcd | bc | cd | bc |
| Oat | bc | d | bc | bc | bc | bc | bc | b | bc | ab |
| Radish | cd | d | c | c | cd | cd | bcd | bc | cd | bcd |
| Canola | cd | cd | bc | c | cd | cd | bcd | bc | cd | cd |
| Rye | d | d | bc | c | cd | e | d | bc | cd | cd |
| 3SppN | bc | bcd | bc | c | cd | bc | b | bc | bc | bc |
| 3SppW | d | d | bc | c | cd | de | d | bc | cd | d |
| 4Spp | d | d | bc | c | cd | de | cd | bc | bcd | bcd |
| 6Spp | d | d | c | c | d | de | d | c | d | cd |
