# Supplementary material for: Managing nitrogen through cover crop species selection in the U.S. mid-Atlantic
Source: PLoS One. 2019 Apr 12;14(4):e0215448. doi: 10.1371/journal.pone.0215448 (PMC6461281; doi:10.1371/journal.pone.0215448)
Supplement: S7 Table — Different letters denote statistical differences among cover crop treatments (rows) for a given time period (columns) in 2014 (October and December) and 2015 (all other dates) based on Fishers LSD and α = 0.05. See Table 1 for treatment codes. (DOCX) [file pone.0215448.s007.docx]

**S7 Table. Statistical results for bucket lysimeter inorganic N data for cover crops grown between maize and soybeans.** Different letters denote statistical differences among cover crop treatments (rows) for a given time period (columns) in 2014 (October and December) and 2015 (all other dates) using Fishers LSD (α = 0.05). See Table 1 for treatment codes.

| Treatment | Season average | Oct 17th | Dec 15th | Dec 18th | Dec 29th | April 6th | April 14th | April 24th |
| --- | --- | --- | --- | --- | --- | --- | --- | --- |
| Fallow | ab | a | ab | ab | abc | ab | abcd | a |
| Pea | a | a | a | a | a | a | a | bc |
| Clover | a | a | ab | a | a | ab | abc | a |
| Oat | bc | a | cd | c | bcd | bc | bcd | a |
| Radish | a | a | abc | abc | bcd | a | ab | a |
| Canola | a | a | ab | abc | ab | a | ab | ab |
| Rye | c | a | cd | bc | bcd | c | e | c |
| 3SppN | c | a | bcd | cd | bcd | bc | cde | c |
| 3SppW | c | a | cd | cd | cd | c | e | c |
| 4Spp | c | a | cd | cd | d | c | de | c |
| 6Spp | c | a | d | d | d | bc | e | c |
