# Supplementary material for: Managing nitrogen through cover crop species selection in the U.S. mid-Atlantic
Source: PLoS One. 2019 Apr 12;14(4):e0215448. doi: 10.1371/journal.pone.0215448 (PMC6461281; doi:10.1371/journal.pone.0215448)
Supplement: S8 Table — Different letters denote statistical differences among cover crop treatments (rows) for a given sampling date (columns) in 2014 based on Fishers LSD and α = 0.05. See Table 1 for treatment codes. (DOCX) [file pone.0215448.s008.docx]

**S8 Table. Statistical results for changes in soil inorganic N, maize leaf tissue N concentration, and maize height in 2014.** Different letters denote statistical differences among cover crop treatments (rows) for a given sampling date (columns) in 2014 using Fishers LSD (α = 0.05). See Table 1 for treatment codes.

|  | % Maize leaf tissue N concentration | | |  | Soil Inorganic N | |  | Maize height | | | |
| --- | --- | --- | --- | --- | --- | --- | --- | --- | --- | --- | --- |
| Treatment | 1 July | 21 Jul | 4 Aug |  | 1 July | 18 Jun 21 Jul 4 Aug |  | 18 Jun | 1 July | 21 Jul | 4 Aug |
| Pea | a | a | a |  | a | * Treatments not significantly different |  | * Treatments not significantly different | ab | a | a |
| Fallow | a | ab | a |  | bc |  |  |  | c | c | b |
| 4Spp | ab | b | ab |  | b |  |  |  | a | ab | ab |
| Rye | b | c | b |  | c |  |  |  | abc | bc | b |
